# Binder-Free Nickel Oxide Lamellar Layer Anchored CoO_x_ Nanoparticles on Nickel Foam for Supercapacitor Electrodes

**DOI:** 10.3390/nano10020194

**Published:** 2020-01-22

**Authors:** Bohua Chen, Yu Zhong, Gengzhe Shen, Fengming Wang, Zhihao Liu, Mei Chen, Weijia Yang, Chi Zhang, Xin He

**Affiliations:** 1 School of Applied Physics and Materials, Wuyi University, Jiangmen 529020, China; cbhmusuia@163.com (B.C.); zhongyuzzby@163.com (Y.Z.); shengzwyu@126.com (G.S.); wuyiwangfengming@126.com (F.W.); wlxylzh607@163.com (Z.L.); chenmei116@126.com (M.C.); yangweijia5377@126.com (W.Y.); ch.zhang@outlook.com (C.Z.); 2 Institute for Mechanical Process Engineering and Mechanics, Karlsruhe Institute of Technology (KIT), 76131 Karlsruhe, Germany

**Keywords:** electrolysis, nickel foam, cobalt oxide, binder-free, supercapacitor

## Abstract

To enhance the connection of electroactive materials/current collector and accelerate the transport efficiency of the electrons, a binder-free electrode composed of nickel oxide anchored CoO_x_ nanoparticles on modified commercial nickel foam (NF) was developed. The nickel oxide layer with lamellar structure which supplied skeleton to load CoO_x_ electroactive materials directly grew on the NF surface, leading to a tight connection between the current collector and electroactive materials. The fabricated electrode exhibits a specific capacitance of 475 F/g at 1 mA/cm^2^. A high capacitance retention of 96% after 3000 cycles is achieved, attributed to the binding improvement at the current collector/electroactive materials interface. Moreover, an asymmetric supercapacitor with an operating voltage window of 1.4 V was assembled using oxidized NF anchored with cobalt oxide as the cathode and activated stainless steel wire mesh as the anode. The device achieves a maximum energy density of 2.43 Wh/kg and power density of 0.18 kW/kg, respectively. The modified NF substrate conducted by a facile and effective electrolysis process, which also could be applied to deposit other electroactive materials for the energy storage devices.

## 1. Introduction

In recent years, energy storage and conversion devices have been attracted great attention to relieve the problems of increasing energy depletion [1,2,3]. Electrochemical devices, such as fuel cells [4], batteries [5], and supercapacitors [6], have proven effective in the practical applications. Among them, supercapacitors are the focus of intense investigations due to their high power density, excellent reversibility, and long cycle life [7,8,9].

According to the charge storage mechanisms of the supercapacitors, they can be classified into two main categories: electric double-layer capacitors (EDLCs) and pseudocapacitors [10,11,12]. Pseudocapacitive materials possess great promise in supercapacitor applications owing to their high theoretical capacitances [13,14,15,16,17,18,19,20,21]. The electrochemical active materials in the electrodes are responsible for charge storage of the devices, while the current collectors take charge in charge transport. Commonly used current collectors include carbon cloth, aluminum foil, and nickel foam (NF) [22,23,24,25]. Among these current collectors, NFs have been advantageous ascribed to their large active surface area and highly conductive 3D network.

The electrodes in energy storage devices are usually fabricated by growing or brushing the electroactive materials on the surface of current collectors via hydrothermal method, electrodeposition, spray pyrolysis, and chemical electrophoresis [7,26,27,28]. To achieve a high ion diffusion and fast faradic reaction, 3D structured active materials are generally designed to boost the specific surface area and active sites [9,29,30,31]. However, the electrode performance is also greatly dependent on the connection between the electroactive materials and current collector because of the great crystal lattice mismatch of the metal nickel substrate/metal compounds, which restricts electron transport capacity and makes electroactive materials separate from the substrate during the long-term cycling process [32,33,34,35,36]. Therefore, it is quite necessary to improve the binding between the electroactive materials and current collector. A tight binding is beneficial to improve the electron transport rate and cycle stability during the repeated charging–discharging process.

Herein, the commercial NF by a facile electrolysis process combined with heating treatment was modified to directly form an oxidation layer with a lamellar structure on the substrate surface. The resulting nickel oxide not only provides a binder-free connection between the current collector and sequent deposited electroactive materials, but also contributes to the capacitive performance of electrode. The distribution and specific surface area of the nickel oxide can be controlled by the electrolytic time and currents. The cobalt oxide nanoparticles could be anchored to the oxidized nickel layer by immersing into the cobalt acetate solution along with a thermal annealing process. The resulting electrode delivers a specific capacitance of 475 F/g at 1 mA/cm^2^, and maintains a high capacitance retention rate of 96% over a cycle life of 3000 times. A simple supercapacitor was assembled to ascertain the practical application of the fabricated electrode, endowing an energy density of 2.43 Wh/kg and a power density of 0.18 kW/kg. The fabrication process of the pseudocapacitive electrode has the advantages of simplicity and low cost, supplying a universal and binder-free electrode substrate for the deposition of other active materials.

## 2. Experimental Section

### 2.1. Preparation of the Oxidized Ni Foam (ONF) and Oxidized Ni Foam Anchored with Cobalt Oxide (ONF/CoO_x_)

Commercial NF with an area of 0.5 cm × 2.5 cm was washed using acetone, deionized water, and ethanol in turn with the assistance of ultrasonic treatment. The cleaned NF substrate was then conducted a typical electrolysis process as a positive electrode by applying a constant current of 0.06 A for 10 min in a direct-current (DC) power system, using a carbon rod as a negative electrode and 0.1 M sulfuric acid (H_2_SO_4_, Xilong Scientific Co., Ltd. AR, Shantou, China, 95–98%) as an electrolyte solution. After that, ONF was obtained by annealing at 100 °C for 1 h in air.

Following this, the as-prepared ONF substrates were immersed into 0.1 M cobalt acetate solution (Co(CH_3_COO)_2_, Macklin, AR, 99.5% of purity), and then placed on a heating plate at 100 °C until drying. The ONF covered with Co^2+^ ions were transferred to an oven at 400 °C for 2 h in air with a temperature rate of 2 °C/min. After heating treatment, the electrode based on the ONF coated with CoO_x_ nanoparticles (ONF/CoO_x_) was achieved. Furthermore, NF/CoO_x_ electrode was also prepared for comparison, by growing CoO_x_ nanoparticles on the NF substrate under the same experimental conditions.

### 2.2. Fabrication of Asymmetric Supercapacitor (ASC) Device

The aqueous ASC device was assembled using the prepared ONF/CoO_x_ electrode as the anode. The cathode was an activated stainless steel wire mesh (SSWM) [37], which was fabricated by a similar electrolysis procedure with the anode. SSWM was activated by applying the electrolysis process with a constant current of 0.06 A for 30 min with 0.1 M sulfuric acid (H_2_SO_4_, Xilong Scientific Co., Ltd. AR, Shantou, China, 95–98%) as the electrolyte solution, and then following annealing treatment at 400 °C for 2 h in air. Moreover, the gel electrolyte in the ASC device consists of 1% poly(vinyl alcohol) (PVA, Macklin, AR, hydrolysis degree of 87–89%) and 1 M potassium hydroxide (KOH, Macklin, SK, Canada, AR, 95% of purity) to avoid leakage. The whole device was sealed in a small glass bottle.

### 2.3. Characterizations and Electrochemical Measurements

The structures and compositions of the electrodes were characterized by Raman spectrometer (Renishaw 2000) and X-ray photoelectron spectroscopy (XPS, ESCALAB 250XI, Thermo Scientific, Waltham, MA, USA) with monochromatic Al Kα radiation. The binding energies were calibrated using the C 1s hydrocarbon peak at 284.6 eV. The surface morphologies of the electrodes were obtained via field emission scanning electron microscopy (FESEM, Sigma 500, Zeiss, Oberkochen, Germany). The microstructure and corresponding mapping analyses of the nanostructures were further observed with transmission electron microscopy (TEM, JEOL2100F).

The electrochemical performance of the electrodes and devices was evaluated on an electrochemical workstation (CHI 760E, CH Instruments Inc., Bee Cave, TX, USA) by cyclic voltammetry (CV), constant-current charge–discharge (GCD) and electrical impedance spectroscopy (EIS). The single electrode was tested with a three-electrode system, and the device was characterized with a two-electrode structure.

## 3. Results and Discussion

**Figure 1:**
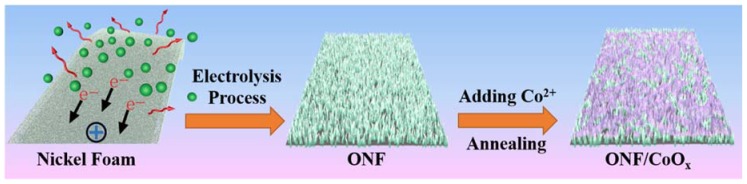
Schematic of the fabrication process of the ONF/CoO_x_ electrodes.

A nanostructured electrode composed of lamellar structured nickel oxide anchored CoO_x_ nanoparticles on the NF substrate was fabricated, as illustrated in Figure 1. The commercial NF substrate was conducted by a facile electrolysis process to obtain an oxidized surface, confirmed by a color change of the foam from silvery-white to dark yellow. Lamellar nanostructures with improved specific surface areas were directly formed on the surface of NF substrate, providing more active sites for the further deposition of active materials. Furthermore, a tight contact between the oxidized nanostructures and the nether Ni layer can be achieved due to the direct oxidation on the foam surface. After immersing the foam in the cobalt acetate solution and then annealing treatment, the rough surfaces were covered by uniformly distributed nanoparticles.

**Figure 2:**
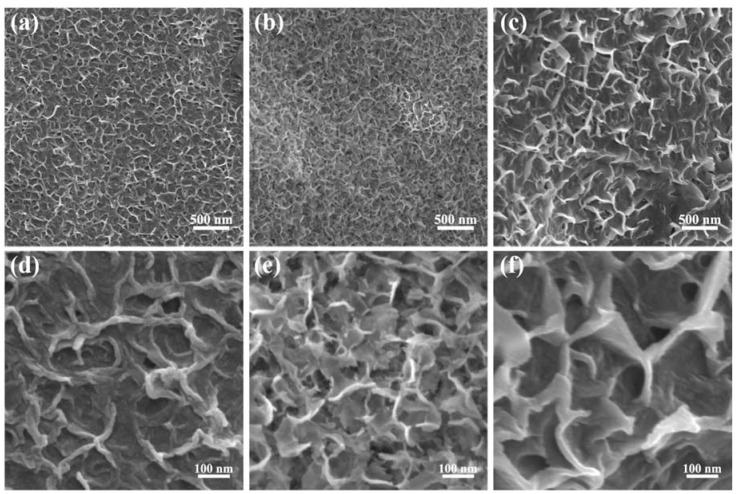
SEM images with a low and high resolution of the NF using electrolysis current of 0.04 (**a**,**d**), 0.06 (**b**,**e**), and 0.08 A (**c**,**f**) for 10 min.

Figure 2 displays low and high magnified SEM images of the ONF electrodes conducted with electrolysis current of 0.04, 0.06, and 0.08 A for 10 min, respectively. The surfaces of NFs after electrolysis have lamellar structures, revealing a great increase in the specific surface area. By comparison, the ONF electrode with an electrolysis current of 0.06 A possesses the densest lamellar-structure, providing an effective skeleton and more active sites for the subsequent growth of active materials.

To obtain an optimized electrolysis current for the ONF electrode, the electrochemical performances of the ONF electrodes with various electrolysis currents were compared. Figure S1 shows the CV curves of the NF substrate and ONF electrodes with different currents. The CV-integrated areas of the ONF electrodes are much larger than that of the pristine NF substrate, suggesting a great enhancement of capacitive performance after the electrolysis process. The oxide layer with the lamellar structure formed on the NF surface could contribute to the electrode capacitance. Moreover, the ONF electrode with the electrolysis current of 0.06 A presents the highest capacitance, due to the largest specific area facilitating the transport of electrolyte ions and speeding up electrochemical reactions, which is consistent with the results of SEM result.

**Figure 3:**
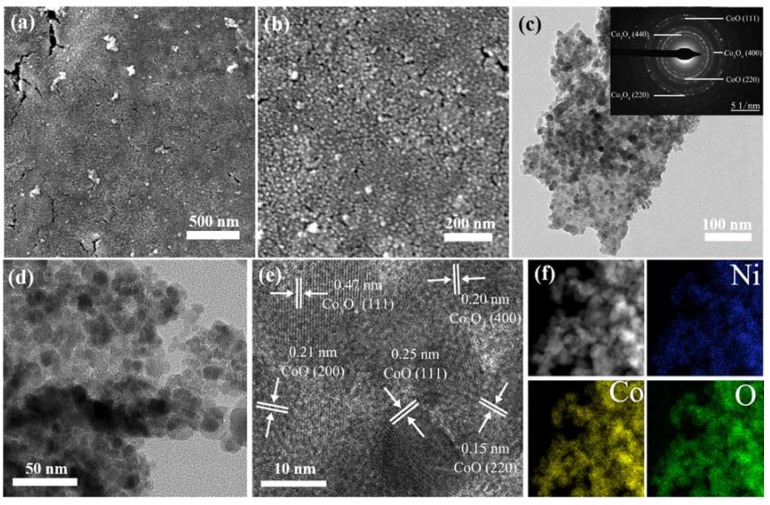
SEM images of ONF/CoO_x_ electrode fabricated using 0.05 M cobalt acetate solution with a low (**a**) and high resolution (**b**), respectively; TEM images of the ONF/CoO_x_ electrode with various magnifications (**c**–**e**); the inset of panel (c) is corresponding SAED pattern; (**f**) the element mapping images of the electrode.

Figure 3a,b shows low and high magnified SEM images of the ONF electrodes coated with nanoparticles. The ONF electrode was fabricated with the current of 0.06 A. After the annealing process, there are nanoparticles with an average size of a dozen nanometers uniformly distributed on the ONF surface. The nanoparticles were anchored to the rough lamellar surface of ONF electrode to form a relatively flat surface.

The morphology and microstructure of the nanoparticles were further analyzed by TEM technique. Figure 3c,d displays low and high magnified TEM images of the sample scraped from the surface of NF substrate, further confirming the formation of nanoparticles. The nanoparticles closely assemble each other, and the individual nanoparticle cannot be observed. The corresponding selected area electron diffraction (SAED) pattern is shown in the inset of Figure 3c, exhibiting concentric diffuse rings and several bright spots. The calculated *d* values from the diffuse rings correspond to the crystal planes of CoO (111), (200), and (220), as well as Co_3_O_4_ (400) and (440). The high-resolution TEM (HRTEM) lattice image is presented in Figure 3e. The continuous lattice fringes reveal a crystal nature of the nanostructures. The distances of plane spacing are measured to be 0.25, 0.21, and 0.15 nm, which agree well with the (111), (200), and (220) lattice planes of CoO. However, the distances are calculated to be 0.47 and 0.20 nm, which is in accordance with the (111) and (400) planes of Co_3_O_4_. No distinct lattice fringes originated from Ni or its oxides can be found. Figure 3f displays the corresponding elemental mapping analyses, manifesting that the element Ni, Co, and O homogeneously distribute throughout the whole sample.

**Figure 4:**
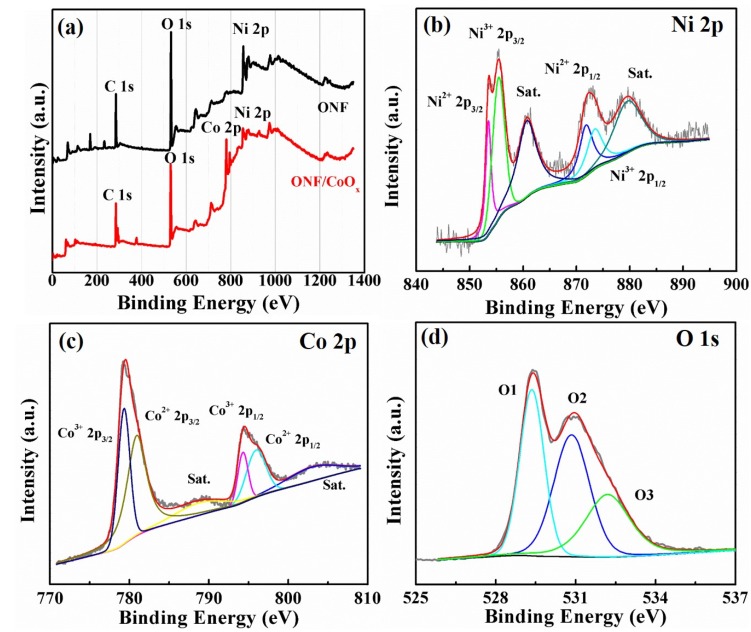
X-ray photoelectron spectroscopy (XPS) spectra of the ONF/CoO_x_ electrode. (**a**) Comparison of ONF and ONF/CoO_x_. High-resolution Ni 2p (**b**), Co 2p (**c**), and O 1s (**d**).

To further identify the chemical element composition and oxidation state of the electrode, XPS measurement was conducted. Figure 4a shows the overall XPS spectra of the ONF and ONF/CoO_x_ electrode, demonstrating the existence of Ni element in the ONF and Ni and Co elements in the ONF/CoO_x_, in addition to the C and O element. The high-resolution XPS spectra of Ni 2p and Co 2p for the ONF/CoO_x_ electrode are depicted in Figure 4b,c. The Ni 2p_3/2_ peak can be decomposed into two fitting peaks located at 853.5 and 855.4 eV, simultaneously accompanied by decomposed Ni 2p_1/2_ peaks at 871.8 and 873.4 eV (Figure 4b), demonstrating that element Ni exists in the lamellar structure in the form of Ni^2+^ and Ni^3+^ with a spin-orbit splitting of 18 and 18.3 eV. The binding energies at 860.7 and 879.5 eV are the two satellite peaks (identified as “Sat.” in Figure 4b,c), which are shake-up-type signals of Ni at the high binding energy [38,39,40]. The bands of Co 2p_3/2_ and Co 2p_1/2_ along with satellite peaks at 788.7 and 803.1 eV in Figure 4c can be deconvoluted into four peaks. The two peaks at 779.4 and 794.3 eV are indexed to Co^3+^, whereas two peaks at 780.9 and 795.9 eV are assigned to Co^2+^ [29]. The distance between Co 2p_3/2_ and Co 2p_1/2_ is 14.9 eV for Co^3+^, while 15 eV for Co^2+^, confirming the presence of cobalt oxide [41]. Figure 4d presents three oxygen peaks contributions of O 1S region of ONF/CoO_x_, which are marked as O1, O2 and O3, respectively. The peak of O1 at 529.4 eV is the typical metal–oxygen bonds, O2 at 531.1 eV comes from oxygen in OH^-^ groups, and O3 at 532.2 eV is attributed to the mixed composition containing Co^2+^, Co^3+^, Ni^2+^ and Ni^3+^ ions [42].

Taking the results of TEM and XPS measurements into considerations, the Ni element in the lamellar structure exists in the form of amorphous nickel oxide, and the Co element on the electrode presents in the form of crystalline CoO and Co_3_O_4_.

**Figure 5:**
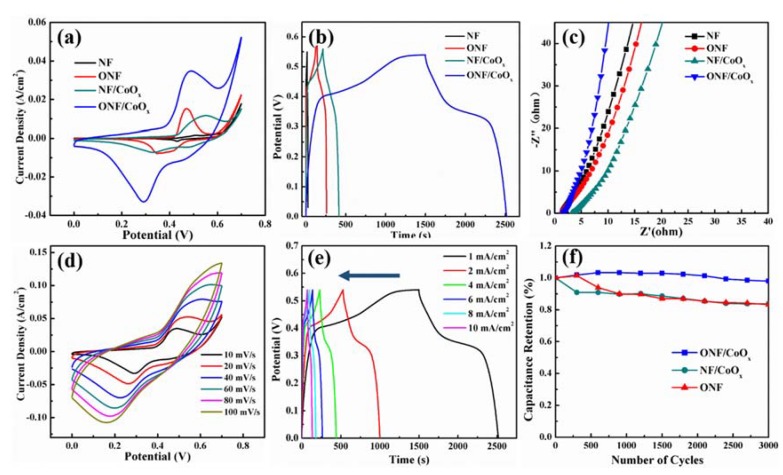
(**a**) CV curves of the NF, ONF, NF/CoO_x_, and ONF/CoO_x_ at 10 mV/s within the potential window of 0–0.7 V; (**b**) GCD curves at 1 mA/cm^2^ and (**c**) Nyquist impedance spectra of four samples; (**d**) CV curves of the ONF/CoO_x_ electrode at the scan rate ranged from 10 to 100 mV/s; (**e**) GCD curves of the ONF/CoO_x_ electrode at the current density ranged from 1 to 10 mA/cm^2^; (**f**) capacitance retention for 3000 cycles of ONF, NF/CoO_x_ and ONF/CoO_x_ electrodes.

To evaluate the electrochemical performance of the ONF/CoO_x_ electrode, electrochemical measurements were made in the three-electrode system with 1 M KOH aqueous solution as an electrolyte, carbon rod as a counter electrode, and Hg/HgO electrode as a reference electrode. Figure 5a compares the CV curves of NF, ONF, NF/CoO_x_, and ONF/CoO_x_ electrodes collected at a scan rate of 10 mV/s. Pristine NF substrate displays a negligible CV-integrated area, revealing the substrate basically does not contribute any capacitive performance. The CV curves of ONF, NF/CoO_x_, and ONF/CoO_x_ electrodes show distinct redox peaks, which are associated with faradic redox reactions related to Ni or/and Co with OH^-^ anions. Notably, the CV-integrated area of the ONF/CoO_x_ electrode is much larger than those of other electrodes, manifesting a great improvement of capacitive performance due to the excellent connection between the current collector and active materials, as well as the synergistic effect of nickel oxide and cobalt oxide [43]. Figure S2 shows CV curves of the ONF/CoO_x_ electrodes using different temperature rates during the heat-treating process, suggesting that the optimized temperature rate is 2 °C/min.

Figure 5b presents the GCD curves of four samples collected at 1 mA/cm^2^. The GCD curves of the ONF, NF/CoO_x_, and ONF/CoO_x_ electrodes reveal a nonlinear pseudocapacitive behavior due to the electrochemical adsorption–desorption or redox reaction at the electrode-electrolyte interface. By comparison, a longer discharge time can clearly be identified for the ONF/CoO_x_ electrode, indicating a higher specific capacity. The mass-specific capacitance of the ONF/CoO_x_ electrode was calculated to be 475 F/g at 1 mA/cm^2^ from GCD curves, which is larger than the recently reported CoO nanoparticles on NF substrate using a binder [44]. Figure 5c plots the EIS spectra of four samples in the frequency range of 1000 kHz to 0.01 Hz. The more vertical straight line along the imaginary Z axis of the ONF/CoO_x_ electrode in the low-frequency regime indicates a lower diffusion resistance and an improved capacitive performance [7,26].

The enhancement of capacitance and rate capability of the ONF/CoO_x_ electrode can be attributed to its unique structural characteristics. First, lamellar-structured nickel oxide can provide large specific area and more active sites for the deposition of CoO_x_ nanoparticles, facilitating the transport of electrolyte ions. Second, CoO_x_ nanoparticles with small sizes promote rapid ion intercalation and delamination owing to enough space among the nanoparticles [45].

Figure 5d,e presents CV curves at various scan rates and GCD curves at various current densities of the ONF/CoO_x_ electrode. The CV curves in Figure 5d show good reversibility as the scan rate elevated from 10 to 100 mV/s. A pair of redox peaks appears in all CV curves, revealing the capacitive characteristic is mainly attributed to the faradic reactions. The oxidation and reduction peaks respectively move slightly to higher and lower potentials as the increase in scan rate [26]. It might be attributed to the larger movement speeds of the OH^-^ at a higher scan rate, generating a faster redox reaction [46]. The GCD curves were measured using current density changed from 1 to 10 mA/cm^2^ within the potential window of 0–0.55 V, exhibiting a pseudocapacitive characteristic with a potential plateau due to the faradic reactions (Figure 5e) [12]. The relationship between the current and specific capacitance/Coulombic efficiency of the ONF/CoO_x_ electrode is plotted in Figure S3, suggesting the highest Coulombic efficiency of 91.6% at the current of 6 mA.

The cycling stabilities of the ONF, NF/CoO_x_, and ONF/CoO_x_ electrodes at 100 mV/s for 3000 charging–discharging cycles are shown in Figure 5f. The ONF/CoO_x_ electrode exhibits highly electrochemical durability with the areal capacitance retention of 96% after 3000 cycles, which is larger than the ONF of 85% and NF/CoO_x_ of 84%. The improved electrochemical stability of the ONF/CoO_x_ electrode originates from the following reason. The nickel oxide directly grown on the NF substrate can anchor the CoO_x_ nanoparticles to its surface, which is confirmed by TEM observation. Thus, both the mechanical strength and adhesion of the active materials to the current collector is greatly increased, which is beneficial for improving the cycling stability.

**Figure 6:**
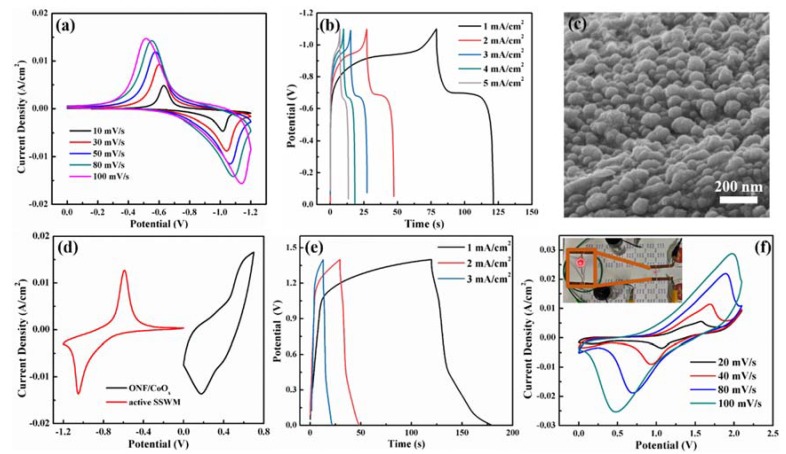
CV curves with various scan rates (**a**). GCD curves with different current densities (**b**) and SEM image (**c**) of the active SSWM electrode. (**d**) CV curves of ONF/CoO_x_ and active SSWM electrode tested at scan rate of 10 mV/s. (**e**) GCD curves of the device obtained at various current densities. (**f**) CV curves of the fabricated ASC device collected at different scan rates, the inset of panel (e) is the photograph of a red LED driven by the devices.

To ascertain the feasibility of the ONF/CoO_x_ electrode in a supercapacitor, an ASC device using the ONF/CoO_x_ as the positive electrode and an activated SSWM electrode as the negative electrode was assembled. Figure 6a,b shows CV curves at different scan rates and GCD curves at various current densities for the activated SSWM electrode, revealing a good capacitive performance and a working voltage window of −1.1 to 0 V. Figure 6c displays the SEM image of the surface morphology of the negative electrode, indicating the rough surface with a relatively high specific area. Figure 6d presents CV curves of ONF/CoO_x_ and activated SSWM electrode tested at 10 mV/s. Figure 6e displays GCD curves of the device at the current densities changed from 1 to 3 mA/cm^2^, confirming good charge transfer and fast charging–discharging capabilities within a potential window of 1.4 V. Moreover, Figure 6f demonstrates the CV curves of the device with scan rates ranging from 20 to 100 mV/s. The symmetric and faradaic redox peaks in all curves reveal typical capacitive characteristics and excellent reaction reversibility.

Additionally, the energy density of the device is calculated to be 2.43 Wh/kg, and the power density is 0.18 kW/kg. A series connection of the two devices is used to light up a red light-emitting diode (LED) with an operating voltage of 1.5 V (the inset of Figure 6f), suggesting its potential applications.

## 4. Conclusions

In conclusion, the ONF/CoO_x_ electrode was fabricated with the assistance of electrolysis and heat treatment. A nickel oxide layer with lamellar structure directly grew on the surface of NF substrate, providing a backbone with a high specific surface area to load active material CoO_x_ nanoparticles. Ascribed to the tight binding between the current collector and electroactive materials, the resultant electrode possesses the specific capacitance of 475 F/g at 1 mA/cm^2^ and the capacitance retention of 96% after 3000 cycles. The assembled asymmetric device based on the ONF/CoO_x_ and activated SSWM yields a wide operation window of 1.4 V, a maximum energy density of 2.43 Wh/kg, and a power density of 0.18 kW/kg. This work should also be applicable to combining other electroactive materials for the fabrication of the binder-free electrodes.

## Supplementary Materials

The following are available online at https://www.mdpi.com/2079-4991/10/2/194/s1, Figure S1: CV curves of the commercial nickel foam and ONFs with various electrolysis currents within the potential window of 0~0.7 V.; Figure S2: CV curves of the ONF/CoOx electrode using different temperature rates during the heat-treating process; Figure S3: Plots of current against the specific capacitance and Coulombic efficiency of the ONF/CoOx electrode.
