# Supplementary material for: Binder-Free Nickel Oxide Lamellar Layer Anchored CoOx Nanoparticles on Nickel Foam for Supercapacitor Electrodes
Source: Nanomaterials (Basel). 2020 Jan 22;10(2):194. doi: 10.3390/nano10020194 (PMC7074865; doi:10.3390/nano10020194)
Supplement: Supplementary file 1 [file nanomaterials-10-00194-s001.pdf]

Supplementary materials

Binder-Free Nickel Oxide Lamellar Layer Anchored CoO_x Nanoparticles on Nickel Foam for Supercapacitor Electrodes

Bohua Chen [1], Yu Zhong [1], Gengzhe Shen [1], Fengming Wang [1], Zhihao Liu [1], Mei Chen [1], Weijia Yang [1], Chi Zhang [1] and Xin He [1,2,*]

[1] School of Applied Physics and Materials, Wuyi University, Jiangmen 529020, China; cbhmusuia@163.com (B.C.); zhongyuzzby@163.com (Y.Z.); shengzwyu@126.com (G.S.); wuyiwangfengming@126.com (F.W.); wlxylzh607@163.com (Z.L.); chenmei116@126.com (M.C.); yangweijia5377@126.com (W.Y.); ch.zhang@outlook.com (C.Z.)

[2] Institute for Mechanical Process Engineering and Mechanics, Karlsruhe Institute of Technology (KIT), 76131 Karlsruhe, Germany

* Correspondence: hexin@mail.wyu.edu.cn

Received: 5 January 2020; Accepted: 20 January 2020; Published: 22 January 2020

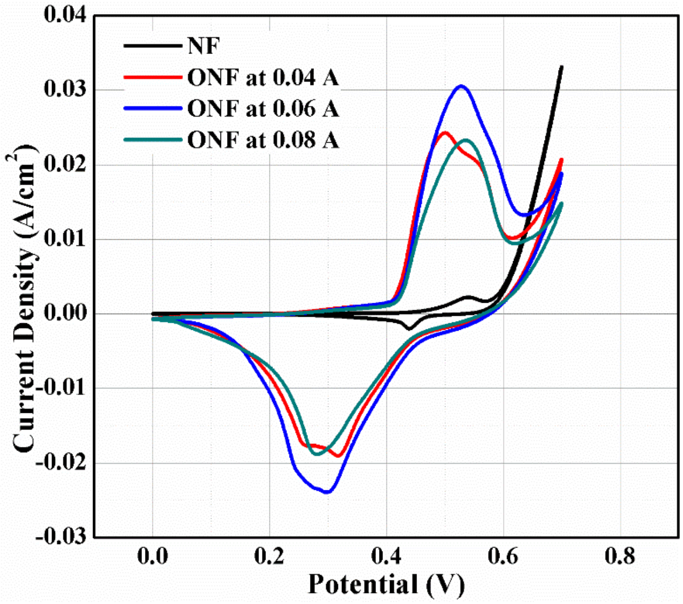

Figure S1: CV curves of the commercial nickel foam and ONFs with various electrolysis currents within the potential window of 0~0.7 V.

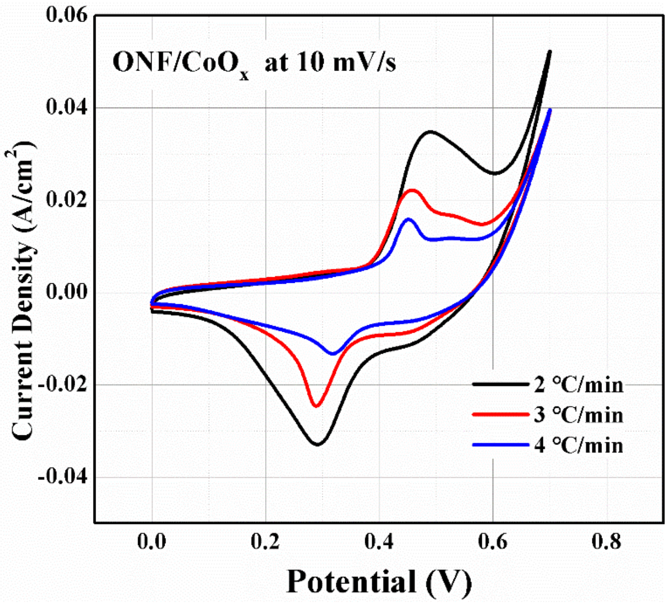

Figure S2: CV curves of the ONF/CoO$_x$ electrode using different temperature rates during the heat-treating process.

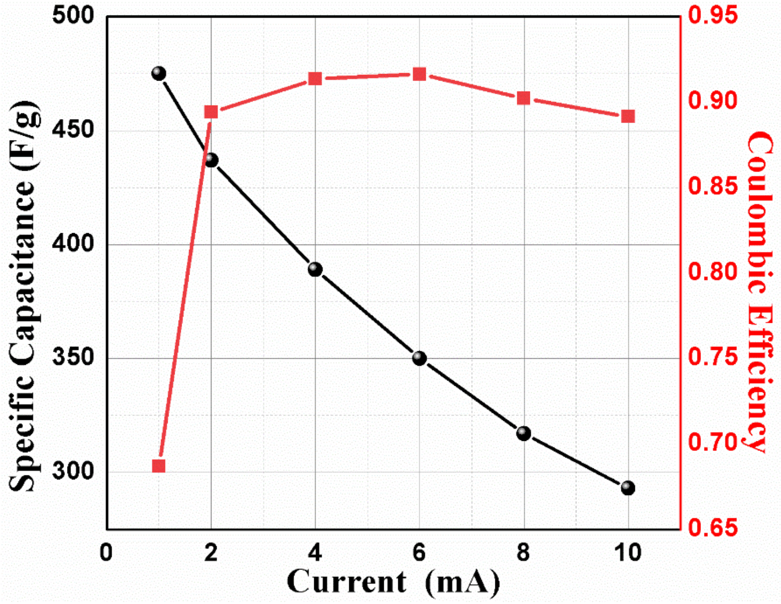

Figure S3: Plots of current against the specific capacitance and Coulombic efficiency of the ONF/CoO$_x$ electrode.
